# Estimation of the spawning time of Japanese eels in the open ocean

**DOI:** 10.1038/s41598-020-61029-8

**Published:** 2020-03-06

**Authors:** Takatoshi Higuchi, Yoshiaki Yamada, Shun Watanabe, Takahito Kojima, Katsumi Tsukamoto

**Affiliations:** 1 Graduate School of Bioresource Sciences, Nihon University, 1866 Kameino, Fujisawa City, Kanagawa 252-0880 Japan; 2 IRAGO Institute Co., Ltd., 377 Ehima-shinden, Tahara, Aichi 441-3605 Japan; 3 Department of Fisheries, Faculty of Agriculture, Kindai University, 3327-204 Nakamachi, Nara, 631-8505 Japan; 4 Department of Marine Science and Resources, Nihon University, 1866 Kameino, Fujisawa City, Kanagawa 252-0880 Japan; 5 Graduate School of Agricultural and Life Sciences, The University of Tokyo, 1-1-1 Yayoi, Bunkyo-ku, Tokyo, 113-8657 Japan

**Keywords:** Fisheries, Theoretical ecology

## Abstract

To understand the spawning ecology of the Japanese eel, the spawning time of this species was estimated based on measurements of the ascending speed of eggs and previously obtained data. Two types of water temperature parameters were calculated assuming an arbitrary spawning time. The ‘incubation temperature’ of 53 eggs collected in the spawning area was estimated based on the developmental stage of each egg and experimentally determined relationships between water temperature and incubation duration. The ‘experienced temperature’ of eggs ascending in the water column after spawning was estimated based on an ascending egg speed of 3.69 m/h and spawning depth of 230 m determined from a pop-up satellite archival tag release experiment on silver eels conducted in the same area. The incubation and experienced temperatures of the eggs coincided only at 20:20–22:30 h, 3 days prior to the new moon. This period is only a few hours after the diel vertical migration of Japanese eels in the evening, when adults move up from a depth of ~800 m (approximately 5 °C) to shallower waters of 200–250 m depth (approximately 20 °C). Our findings will facilitate improvements in aquaculture techniques and the detection of eel spawning events in the open ocean.

## Introduction

An exponential decrease in freshwater eel resources began in the late 1900s^1^. With less than 1% of eel resources remaining in the Atlantic, the International Union for the Conservation of Nature and Natural Resources (IUCN) decided to list four anguillid eel species, namely the European eel *Anguilla anguilla*, American eel *A. rostrata*, Japanese eel *A. japonica*, and Borneo eel *A. borneensis*, as threatened species in 2014^2^. Several factors have been suggested to have contributed to the decline in eel resources, including overfishing and the destruction of estuarine environments and freshwater habitats^3,4^. Environmental fluctuations in oceanic spawning habitats and migration pathways may also have influenced the fluctuation in freshwater eel resources^4–8^.

In order to gain an understanding of the mechanisms underlying the fluctuations in eel resources, the entire enigmatic reproductive ecology of anguillid eels must be studied in detail. In 2008 and 2009, spawning adults and eggs of the Japanese eel were respectively collected from the west side of the Mariana Islands^9–11^, revealing the existence of Japanese eel spawning sites near the southern end of the West Mariana Ridge in the Philippine Sea^10,12^. However, the courtship behaviour of these freshwater eels and the structure of spawning populations must also be characterised in order for us to fully comprehend their spawning ecology. Direct observation of the spawning behaviour of adult eels in the field is the obvious way to achieve this objective. However, although various efforts involving the manned submersible JAGO (Max Planck Institute) and SHINKAI 6500 (JAMSTEC), the underwater survey system Deep-Tow (JAMSTEC), and the UNA-CAM drifting camera systems (JAMSTEC and Nihon University) have attempted to observe spawning events^13,14^, the tracking of spawning eels has yet to be accomplished. To solve this problem, novel methods that can be used to estimate the precise location and timing of spawning events are essential.

Recently, attempts have been made to identify the location of spawning areas in studies using a prediction model based on the ‘Internal Tide Hypothesis’ (Higuchi *et al*., unpublished). In brief, adult eels might spawn in high-energy patches of internal tide in the eels’ putative eel spawning area. Studies that have examined the otolith microstructure of Japanese eel larvae suggest that spawning occurs during the new moon period of each month in summer^15,16^. Moreover, the results of numerous studies on the eggs and hatched larvae of Japanese eels have clearly indicated that spawning events occur a few days before the new moon^10,12^. Nevertheless, further detailed estimations of the time of spawning are needed to locate the aggregations of spawning adult eels.

Thus, the objective of the present study was to estimate the spawning time of Japanese eels using two different types of water temperature measurement: (1) the incubation temperature, estimated based on the embryonic developmental stage of each collected egg; and (2) the experienced temperature, calculated based on the speed of eggs moving up the water column (hereafter referred to as the ascent speed) and the recorded ocean water temperature. The time of spawning is herein considered to be the period during which these two temperatures coincide. Our findings not only can be used to identify the spawning events of freshwater eels in the ocean but also may contribute to the formulation of resource management plans and development of aquacultural procedures for freshwater eels, which, despite substantial efforts for over half a century, have yet to be established.

## Results

### Estimation of spawning time

**Figure 1:**
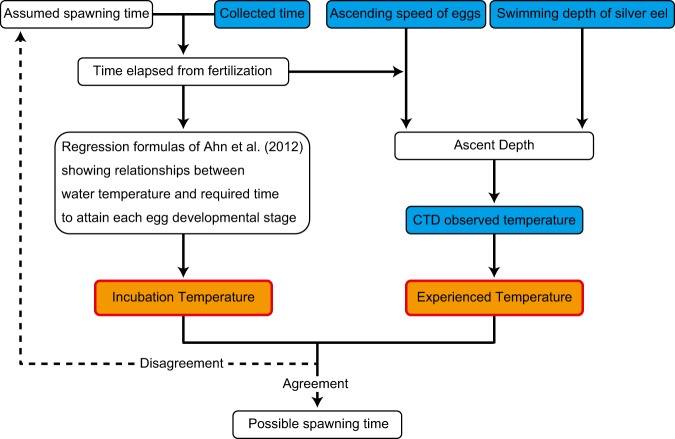
Method used to estimate the spawning time of Japanese eels. Egg collection times (Supplementary Fig. S4)^10,12^, ascending speed of eggs (Fig. 2), swimming depth of silver eels (Supplementary Fig. S2)^18^, and Conductivity Temperature Depth profiler observed water temperatures are indicated by blue boxes. The assumed spawning time (overlap of the incubation and experienced temperatures, indicated by orange boxes) is considered to be the possible spawning time.

In this study, we used two types of environmental water temperature parameters experienced by the eggs to estimate spawning time, namely the incubation temperature and the experienced temperature (Fig. 1). Arbitrary spawning time (*AST*) was assumed at any time before the new moon, and the time elapsed from fertilisation (*ET*, unit: hour) until egg collection (*EC*, Supplementary Fig. S1) was calculated for each egg (Eq. 1).

1 $$ET=EC-AST$$

For example, if the arbitrary spawning time is assumed at 21:00 on 3 days prior to the new moon for one fertilized egg that is collected at 3:00 on 1 day prior to the new moon, the time elapsed from fertilisation is calculated to be 30 hours.

The experienced temperature (unit: °C) is the temperature experienced by an egg prior to collection. This temperature is defined as the mean water temperature experienced by each egg during its ascent from the depth at which spawning occurred to the depth at which the egg ascent. Based on spawning depth (*SD*, unit: m), ascending speed (*AS*, unit: m h^−1^) and *ET*, the ascent depth (*AD*_*ET*_, unit: m) was calculated (Eq. 2).

2 $$A{D}_{ET}=SD-AS\times ET$$

The rising arrival depth is deeper as the elapsed time from fertilisation is shorter. If the eggs reached the upper border of the pycnocline near the 150 m layer in the spawning area^12^, it was assumed that the eggs would stop rising at 150 m in the water column. The experienced temperature is the averaged value of the water temperature that eggs experienced during the ascending process (Eq. 3). The water temperature is the observed water temperature (*Temp*_*n*_, unit: °C) at corresponding depth at which the egg was present throughout the ascending process (*AD*_*n*_, unit: m).

3 $$Experienced\,temperature=\frac{1}{ET}\mathop{\sum }\limits_{n=1}^{ET}(Tem{p}_{A{D}_{n}})$$

The calculated experienced temperature is lower as the elapsed time from fertilisation is shorter.

The incubation temperature (unit: °C) is defined as the water temperature at which the collected egg reached a certain developmental stage. On the basis of the relational equations between experienced temperature (variable *x*) and time required for an artificially fertilised egg to reach a certain developmental stage (variable *y*), as reported by Ahn *et al*.^17^, incubation temperature values were obtained by assigning *ET* to the variable *y* in the relational equation^17^. For eggs collected at different stages, the relational formulas reported by Ahn *et al*.^17^ corresponding to each developmental stage were used. (Eq. 4 for eggs at blastula stage, Eq. 5 for eggs at gastrula stage, Eq. 6 for eggs at eye and ear vesicle formation stage, Eq. 7 for eggs at heart formation stage, Eq. 8 for eggs at pre-hatching stage)

4 $$Incubation\,temperature\,(blastula)=\frac{ET-11.39}{-0.2233}$$

5 $$Incubation\,temperature\,(gastrula)=\frac{ET-15.383}{-0.3}$$

6 $$Incubation\,temperature\,(eye\,and\,ear\,vesicle\,formation)=\frac{ET-67.822}{-1.9889}$$

7 $$Incubation\,temperature\,(heart\,formation)=\frac{ET-2.5222}{-88.689}$$

8 $$Incubation\,temperature\,(pre\,hatching)=\frac{ET-102.52}{-2.8556}$$

Thus, eggs at different stages collected at the same sampling station had different incubation temperatures. Because the egg collection time is fixed, the time elapsed from fertilisation until egg collection is variable by assumed spawning time. So, the elapsed time from fertilisation is shorter as the assumed spawning time is later, and the calculated incubation temperature is higher as the elapsed time from fertilisation is shorter.

If the assumed spawning time was the actual spawning time, the incubation and experienced temperatures were expected to coincide. Therefore, the overlap between these two estimated temperatures is herein considered to indicate the time of spawning.

### The ascending speed of eggs

**Figure 2:**
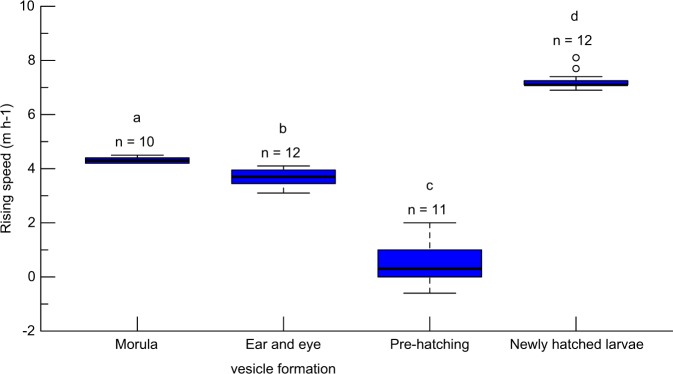
Box plots showing a comparison of ascending speeds among eggs at the morula, ear and eye vesicle formation, and immediately pre-hatching stages, and of newly hatched larvae of Japanese eels. The numbers above each box plot indicate the sample sizes for each developmental stage. Different letters above the boxes indicate a significant difference.

By observing artificially spawned eggs and their resultant preleptocephali in a cylindrical column tank, we determined the ascending speeds of eggs at the morula stage, ear and eye vesicle formation stage, and immediately prior to hatching, and of newly hatched larvae as 4.32 ± 0.11 (4.2–4.5), 3.69 ± 0.31 (3.1–4.1), 0.49 ± 0.72 (−0.6–2.0), and 7.24 ± 0.32 m h^−1^ (6.9–8.1 m h^−1^), respectively (Fig. 2). Statistical analyses revealed that the speeds of ascent differed significantly among the different developmental stages (*p* < 0.001, Kruskal–Wallis with Steel–Dwass post hoc test), indicating that the embryonic buoyancy decreases gradually with development and then increases rapidly after hatching^18^.

Eggs at the morula stage, shortly after fertilisation, may experience a temperature of under 20 °C at the estimated spawning depth (230 m) in their spawning area^12^, which was tracked using pop-up satellite archival tags^19^ (Supplementary Fig. S2). It is also known that eggs and newly hatched larvae accumulate at the thermocline (at a depth of approximately 150 m), at which the water temperature is approximately 26 °C, since the upper layer is warmer and lighter, and the eggs and larvae are unable to rise further in the water column^10,12^. Thus, eggs and early larvae may experience water temperatures of at least 20–26 °C. Most of the eggs collected during this study were at the late stage of development immediately prior to hatching, the measured ascending speed of which was between −0.6 and 4.5 m h^−1^. We used the value of an intermediate stage (3.69 m h^−1^; ear and eye vesicle formation stage) as the representative ascending speed of eggs in the ocean to estimate the ascent depth of the fertilised eggs.

### Spawning date

The developmental stages of only 53 of the 593 eggs collected during the four *R/V* Hakuho Maru cruises from 2009 to 2012 were determined, as the remaining 540 eggs were either non-viable or damaged, or were unclearly photographed (photographs were taken only after a rapid onboard morphological identification) (Supplementary Fig. S3). Among the remaining 53 eggs, 7, 9, and 37 were at the ear and eye vesicle formation, heart formation stage, and immediately pre-hatching stages, respectively.

**Figure 3:**
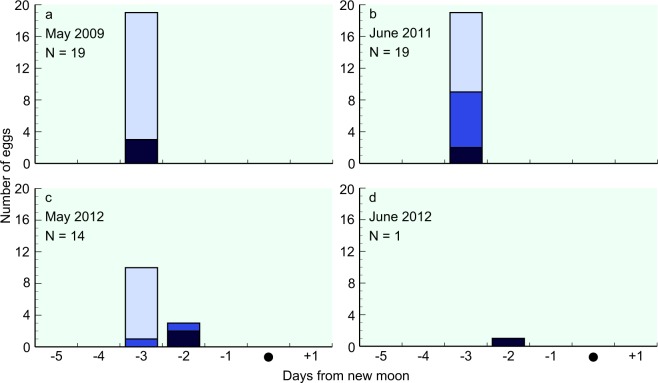
Estimated spawning date in relation to the new moon during each cruise period. The determined developmental stages of egg are as follows: ear and eye vesicle formation stage (dark blue bars), heart formation stage (blue bars), and immediately pre-hatching (light blue bars). All eggs collected are plotted in relation to the date of the new moon of each month: (**a**) May 2009, (**b**) June 2011, (**c**) May 2012, and (**d**) June 2012.

In May 2009, we collected three eggs at the ear and eye vesicle formation stage on days 1 and 2 before the new moon, and 16 eggs at the immediately pre-hatching stage on day 2 before the new moon (Supplementary Fig. S4). Using the formula described by Ahn *et al*.^17^, their spawning dates were estimated to be day 3 before the new moon (21^st^ May 2009) (Fig. 3). Similarly, in June 2011, 2, 7, and 10 eggs at the ear and eye vesicle formation, heart formation, and immediately pre-hatching stages, respectively, were collected on day 1 before the new moon (Supplementary Fig. S4), all of which were estimated to have been spawned on day 3 before the new moon (27^th^ May 2011). On 29^th^ June 2011, eggs at the heart formation stage were collected at 12°54′N, 141°55′E at 04:29 h (Supplementary Fig. S3), and dead eggs were collected at 13°5′N 142°5′E at 21:32 h (Supplementary Fig. S3). The dead eggs were undeveloped although not putrid, and were thus assumed to have been spawned in the same event as the eggs collected at the heart formation stage.

In May 2012, we collected 1, 2, and 11, eggs at the ear and eye vesicle formation, heart formation, and immediately pre-hatching stages. Their spawning dates were estimated to be on day 3 (17^th^ May 2012) or day 2 (18^th^ May 2012) before the new moon. One egg at the heart formation stage and 11 eggs at the immediately pre-hatching stage were collected at 15°5′N 142°20′E in the morning (07:46 h) on 19^th^ May 2012, and on 20^th^ May 2012, one egg at the ear and eye vesicle formation stage and one egg at the heart formation stage were collected at night (01:43–04:30 h) at almost the same location (15°5′N, 142°20′E and 15°N, 142°20′E, respectively). Given that the older hatching eggs (Supplementary Fig. S3) were collected approximately 26 h prior to collection of the younger eggs at the ear and eye vesicle formation stage (Supplementary Fig. S3) at virtually identical sites, we can assume that multiple spawning events occurred on consecutive nights within the same area. In June 2012, one egg at the ear and eye vesicle formation stage was estimated to have been spawned on day 2 before the new moon (17^th^ June 2012). Accordingly, on the basis of the distribution of the estimated spawning dates, we can assume that spawning (94.5%) occurred on day 3 before the new moon in each spawning month.

### Spawning time

**Figure 4:**
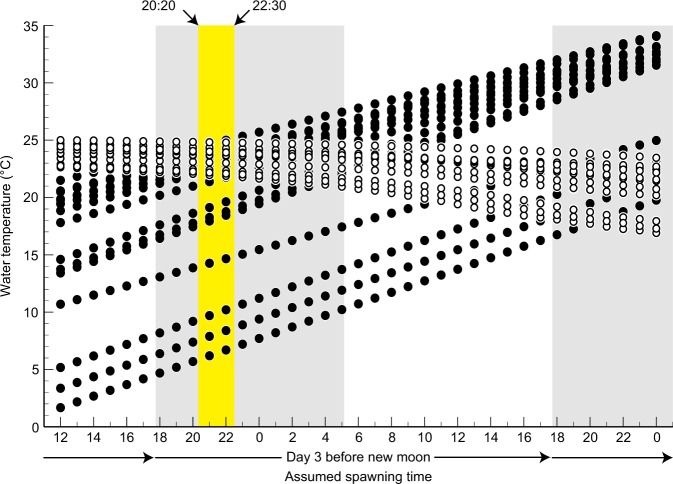
Estimated spawning time on days 2 to 4 before the new moon. The experienced temperature calculated by Eq. 3 (white circles) and incubation temperature calculated by Eqs. 4–8 (black circles) for 53 of all 593 eggs at each assumed spawning time (horizontal axis), estimated following the method shown in Fig. 1. Comparison of the two types of water temperatures that are plotted above each assumed spawning time showed no significant difference from 20:20 to 22:30 h on day 3 before the new moon (*p* > 0.05, Brunner–Munzel test) (yellow area). The grey-shaded areas show the time between sunset and sunrise, which is averaged among the four research cruises.

A comparison between the incubation and experienced temperatures for each assumed spawning time indicated that there was no significant difference between these temperatures only between 20:20 and 22:30 on day 3 before the new moon, when the mean incubation and experienced temperatures for n = 53 eggs were 22.07 ± 3.94 °C and 23.21 ± 0.95 °C, respectively (Brunner–Munzel test, *p* > 0.05) (Fig. 4). Further, incubation and experienced temperatures of 4 of 53 eggs overlapped on day 2 before the new moon (Fig. 3). On the basis of this observation, we thus predict that the spawning time of the Japanese eel coincides with this time window. The 53 eggs that were used for estimation of spawning time were collected from 21:00 h to 11:00 h on days 3 to 1 before the new moon. Most of the eggs (69.81%) were collected just after the peak of spawning time (20:20–22:30 h on day 3 before the new moon) (Supplementary Fig. S1).

## Discussion

The most significant aspect of the present study is that we present the first estimate of eel spawning time based on data of Japanese eel eggs recently collected in their natural environment. Moreover, to the best of our knowledge, we performed the first experimental measurements of the speeds of eggs ascending in a water column. We thus believe that the findings of the present study may have unravelled at least some of the mystery surrounding the spawning ecology of freshwater eels. The newly estimated spawning time between 20:20 and 22:30 h on day 3 before the new moon falls within previous estimates of the spawning period (days 2–4 before the new moon)^15,16^. And the two types of estimated water temperatures were consistent with previous studies that are the optimal temperature for spawning behavior of spawning-condition eels (22 °C)^20^ and eggs development/hatching (22–25 °C)^17^. On the basis of these findings, we infer that adult males and females gather in the evening and commence spawning courtship activities, thus spawning in the aforementioned time window (Yamada *et al*. personal communication).

The estimated spawning time (approximately 21:30 h on day 3 before the new moon) is only a few hours after Japanese eels ascend from the deep layer during their diel vertical migration (DVM) (Supplementary Fig. S2). In both their spawning area and along migration routes after leaving their freshwater growth habitat, Japanese eels repeatedly perform a unique daily vertical movement between deep/cold (787.6 ± 54.9 m, 5.2 ± 0.3 °C) and shallow/warm (267.3 ± 52.6 m, 18.2 ± 3.0 °C) water (Supplementary Fig. S2)^19^. The timing of their ascent through the water column coincides with sunset and all silver eels in the same area generally ascend at the same time^21^. It is thus conjectured that this DVM may play a role in the physiological synchronisation of final maturation.

The estimated spawning time also coincides with low tide and the ensuing flood tide (eastward tidal velocity varied from −3.30 ± 0.58 to 3.14 ± 0.53 cm s^−1^)^22^. The reproduction of marine organisms, such as white-streaked grouper, *Epinephelus ongus*^23^ and Zebra coral, *Oulastrea crispata*^24^, is often synchronised with periods of slack tide. This may have certain ecological advantages, in that slack water may promote fertilisation success, and the ensuing flood flow can facilitate the effective dispersal of eggs, thereby minimizing the likelihood of predation. In the pelagic region, adult eels are the common targets of visually hunting predators with high swimming performance, including tunas, sharks, and marine mammals^25–27^, and thus the spawning of Japanese eels on the nights of a new moon may lessen the risk of falling prey to these predators.

The spawning depth of Japanese eel (230 m) is estimated by the nighttime depth (Supplementary Fig. S2). Higuchi *et al*. (2018)^19^ reported that Japanese eels distribute on stable layer (230.2 ± 20.0 m) when the moon disappeared in the sky during nighttime. Furthermore, it has been clarified that the swimming depth during their spawning migration is regulated by light environment from the several pop-up satellite archival tag studies^18,28–30^ and pinger tracking study^31^. Japanese eels that arrive at same spawning site may swim almost same layer. Therefore, the swimming depth during nighttime might align the spawning depth and increase the probability for spawning-condition Japanese eels to encounter another spawning-condition adults.

For a number of years, considerable efforts have been made in order to establish aquacultural procedures for anguillid eels. To date, however, artificial maturation, spawning, and fertilisation through exogenous hormone treatment have been applied for only five of the 19 known species/subspecies of anguillid eels worldwide, namely, *A. japonica*^32^, *A. anguilla*^33,34^, *A. rostrata*^35^, *A. australis*, and *A. dieffenbachii*^36^. However, with the exception of some incomplete investigations using high water pressure^37^ and fluctuating water temperature manipulation^38^, there have been no investigations based on the natural progress of sexual maturation without the use of exogenous hormone treatments. Accordingly, the natural process of final maturation for spawning remains incompletely understood. Because the estimated timing of spawning event is just after ascending of DVM (Supplementary Fig. S2a,b), increasing of the experienced water temperature and water pressure might be one of the stimuli for start of ovulation and spawning. The spawning events on 2–3 days prior to the new moon (Fig. 3) is equivalent to 27–28 of moon age. Since eels experience a gradually darkening night as they approach the new moon, light environment may be a supplemental stimulus.

In order to understand all aspects of the spawning ecology of freshwater eels in their natural environment, the Internal Tide Hypothesis, which proposes that male and female eels use the high energy of internal tides for spawning aggregation and mating, must be validated by observing spawning behaviour in their spawning area^13,14^. The information on spawning time presented in this study will contribute to predicting the spatiotemporal position of these fish and in detecting their spawning events.

If the spawning behaviour can be observed directly, several ecological and biological mysteries, including the number of spawners, sex ratio, and courtship, may be resolved. Such knowledge will provide a scientific basis for devising effective measures for the resource management, propagation, and conservation of eels. Moreover, our proposed scenario for final maturation in the natural environment may contribute to enhancing aquaculture procedures for promoting spontaneous maturation.

In conclusion, the present study proposed a new method for estimation of the spawning time of Japanese eels using results from four survey cruises, experimental data of the ascending speed of eggs, and the swimming depth of a silver eel in its spawning area. To improve the accuracy of the estimation, some factors must be optimised. The spawning depth was estimated to be 230 m based on the DVM behaviour of Japanese eel^19^. This depth should be confirmed by observing the spawning-condition adults on the west side of the Mariana Islands. Furthermore, measurement of the ascending speed of eggs was conducted using seawater with stable temperature and salinity. However, the environmental conditions of a water column in ocean water vary according to depth. To better simulate actual conditions, similar experiments using various temperatures and salinities should be conducted to generate a more precise model of ascension.

## Methods

### Collection of japanese eel eggs

A total of 593 Japanese eel eggs were collected during four research cruises of the R/V Hakuho Maru in May 2009, June 2011, and May and June 2012. These eggs were collected using the methodology described by Tsukamoto *et al*.^10^ and Aoyama *et al*.^12^. In brief, eggs were caught using standardised oblique tows of a 3-m diameter ORI-BigFish ring net with 0.5-mm mesh that fished mostly in the upper 200 m. The collected eggs were identified both morphologically and genetically^39–41^. The position of egg collection was reported by Tsukamoto *et al*.^10^ and Aoyama *et al*.^12^. In the present study, a unit of day was regarded as the 24-h period from one sunset to the next, and the new moon was considered as ‘day 0’.

In May 2009, 31 eggs were collected on days 2 to 1 before the new moon^10^ (Supplementary Fig. S4). A total of 147 eggs were also collected on days 3 to 2 before the new moon in June 2011^12^. In May 2012, 131 eggs were collected on days 2 to 0 before the new moon, whereas in June 2012, 284 eggs were collected on days 3 to 1 before the new moon. Egg collection occurred on day 2 before the new moon during each survey cruise.

### Measurement of the ascending speed of japanese eel eggs

The speed at which the eggs/larvae of Japanese eel ascend in a water column was measured in the laboratory. The eggs used in this study were obtained from the spontaneous spawning of artificially matured Japanese eels (three males and one female). The three males were collected during the glass eel stage and reared at the IRAGO Institute, Aichi, Japan. The female originated from artificial seedling production and was reared at the same institute as the three males. These four eels were artificially matured following the method reported by Kagawa *et al*.^42^ and Okamura *et al*.^43,44^. The fertilised eggs were incubated in seawater at 23–25 °C until they reached each of the following developmental stages: morula, ear and eye vesicle formation, immediately pre-hatching, and newly hatched larvae.

Analysis of ascending eggs/larvae was conducted 10 or 11 times for each developmental stage using a 27-L transparent tank (width 30 cm × depth 10 cm × height 90 cm) filled with seawater (temperature: 20 °C, salinity: 35‰). The condition of seawater, including salinity and temperature, was controlled to roughly mimic the environmental factors of the estimated spawning layer (about 230 m in present study) observed in the survey in June 2011 that was reported in Aoyama *et al*.^12^ and to prevent effects on normal embryonic development. The eggs and larvae were gently released near the bottom of the tank, and the speeds of ascent were measured using a stopwatch. The ascent speeds were compared statistically using the Kruskal–Wallis test followed by Steel–Dwass multiple comparisons.

The handling of adult eels and measuring of egg ascending were carried out in strict accordance with the relevant guidelines and regulations, and our protocol was approved by Institutional guidelines for animal experiments of Nihon University. All eels were anaesthetized using 2–phenoxyethanol before handling.

### Developmental stage determination and water temperature estimation of natural eggs

The developmental stages of eggs collected in the spawning area were determined following Yamamoto *et al*.^45^.

Spawning depth was estimated based on pop-up satellite archival tag data of a Japanese eel released in its spawning area (Supplementary Fig. S2)^18^. Spawning depth was assumed to be 230 m since the mean ± standard deviation of swimming depths at the peak of egg collection (2 days before the new moon) was 229.2 ± 9.9 m. The ascent depth of eggs (the depth at which an egg with positive buoyancy ascended in the ocean) was estimated based on the measured ascending speed of eggs, the estimated spawning depth, and the time elapsed since fertilisation.

On the basis of the vertical structure of water temperature estimated using a conductivity, temperature, and depth (CTD) sensor system (Sea-Bird, USA), data for the experienced temperature (overall temperature experienced during the ascent of each collected egg) were calculated as the mean of the temperatures recorded for each 1-m interval from a depth of 230 m to the ascent depth of each egg. Estimates of the spawning time of the Japanese eels were obtained as the time window represented by the statistically non-significant difference between incubation and experienced temperatures based on the Brunner–Munzel test (Fig. 1).

## Supplementary information

Supplementary information.

## Data Availability

This study was carried out using previously published data^10,12,18^ (*e.g*. information about collected eggs, tracking data of an adult eel, and environmental information regarding the research area of this study), excluding those described in ‘Measurement of the ascending speed of Japanese eel eggs’.

## References

[1] Dekker W, Casselman JM (2014). The 2003 Québec Declaration of Concern About Eel Declines—11 Years Later: Are Eels Climbing Back up the Slippery Slope?. Fisheries.

[2] Jacoby DMP (2015). Synergistic patterns of threat and the challenges facing global anguillid eel conservation. Glob. Ecol. Conserv..

[3] Dekker, W. Slipping through our hands Population dynamics of the European eel (2004).

[4] Tsukamoto K, Aoyama J, Miller MJ (2009). Present status of the Japanese eel: resources and recent research. in Eels at the edge: Science, status, and conservation concerns. American Fisheries Society Symposium.

[5] Kim H (2007). Effect of El Niño on migration and larval transport of the Japanese eel (*Anguilla japonica*). ICES J. Mar. Sci..

[6] Zenimoto K (2009). The effects of seasonal and interannual variability of oceanic structure in the western Pacific North Equatorial Current on larval transport of the Japanese eel *Anguilla japonica*. J. Fish Biol..

[7] Chang YL, Sheng J, Ohashi K, Béguer-Pon M, Miyazawa Y (2015). Impacts of interannual ocean circulation variability on Japanese eel larval migration in the western north pacific ocean. PLoS One.

[8] Chang YL, Miyazawa Y, Béguer-Pon M (2016). Simulating the Oceanic Migration of Silver Japanese Eels. PLoS One.

[9] Chow S (2009). Discovery of mature freshwater eels in the open ocean. Fish. Sci..

[10] Tsukamoto, K. *et al*. Oceanic spawning ecology of freshwater eels in the western North Pacific. *Nat. Commun*. **2**; 10.1038/ncomms1174 (2011). 10.1038/ncomms1174 PMC3105336 21285957

[11] Kurogi H (2011). First capture of post-spawning female of the Japanese eel *Anguilla japonica* at the southern West Mariana Ridge. Fish. Sci..

[12] Aoyama J (2014). Spawning sites of the Japanese eel in relation to oceanographic structure and the West Mariana Ridge. PLoS One.

[13] Tsukamoto K (2013). Video observation of an eel in the *Anguilla japonica* spawning area along the West Mariana Ridge. Fish. Sci..

[14] Fukuba T (2015). A new drifting underwater camera system for observing spawning Japanese eels in the epipelagic zone along the West Mariana Ridge. Fish. Sci..

[15] Ishikawa S (2001). Spawning time and place of the Japanese eel *Anguilla japonica* in the North Equatorial current of the western North Pacific Ocean. Fish. Sci..

[16] Tsukamoto K (2003). Seamounts, new moon and eel spawning: The search for the spawning site of the Japanese eel. Environ. Biol. Fishes.

[17] Ahn H (2012). Effect of water temperature on embryonic development and hatching time of the Japanese eel *Anguilla japonica*. Aquaculture.

[18] Higuchi, T. *et al*. Tracking *Anguilla japonica* silver eels along the West Mariana ridge using pop-up archival transmitting tags. *Zool. Stud*. **57**; 10.6620/ZS.2018.57-24 (2018). 10.6620/ZS.2018.57-24 PMC6517768 31966264

[19] Tsukamoto K (2009). Positive buoyancy in eel leptocephali: An adaptation for life in the ocean surface layer. Mar. Biol..

[20] Dou SZ (2008). Temperature influence on the spawning performance of artificially-matured Japanese eel, *Anguilla japonica*, in captivity. Environ. Biol. Fishes.

[21] Chow S (2015). Light-sensitive vertical migration of the Japanese eel *Anguilla japonica* revealed by real-time tracking and its utilization for geolocation. PLoS One.

[22] Niwa Y, Hibiya T (2014). Generation of baroclinic tide energy in a global three-dimensional numerical model with different spatial grid resolutions. Ocean Model..

[23] Nanami A, Sato T, Ohta I, Akita Y, Suzuki N (2013). Preliminary observations of spawning behavior of white-streaked grouper (*Epinephelus ongus*) in an Okinawan coral reef. Ichthyol. Res..

[24] Zayasu Y, Miyazaki K, Lien YT, Okubo N (2015). Direct evidence of sexual reproduction in the zebra coral, *Oulastrea crispata* (Anthozoa, Scleractinia), in Japan. Invertebr. Reprod. Dev..

[25] Béguer-Pon M (2012). Shark Predation on Migrating Adult American Eels (*Anguilla rostrata*) in the Gulf of St. Lawrence. PLoS One.

[26] Béguer-Pon, M., Castonguay, M., Shan, S., Benchetrit, J. & Dodson, J. J. Direct observations of American eels migrating across the continental shelf to the Sargasso Sea. *Nat. Commun*. **6**; 10.1038/ncomms9705 (2015). 10.1038/ncomms9705 PMC4918406 26505325

[27] Wahlberg M (2014). Evidence of marine mammal predation of the European eel (*Anguilla anguilla* L.) on its marine migration. Deep. Res. Part I Oceanogr. Res. Pap..

[28] Schabetsberger R (2013). Oceanic migration behaviour of tropical pacific eels from Vanuatu. Mar. Ecol. Prog. Ser..

[29] Chen S, Chang C, Han Y (2018). Seaward Migration Routes of Indigenous Eels, *Anguilla japonica, A. marmorata*, and *A. bicolor pacica*, via Satellite Tags. Zool. Stud..

[30] Watanabe, S. *et al*. Reexamination of the spawning migration of *Anguilla dieffenbachii* in relation to water temperature and the lunar cycle. New Zeal. *J. Mar. Freshw. Res*. 1–17 (2019).

[31] Chow S (2015). Light-sensitive vertical migration of the Japanese eel *Anguilla japonica* revealed by real-time tracking and its utilization for geolocation. PLoS One.

[32] Yamamoto K, Yamauchi K (1974). Sexual maturation and production of Japanese eel larvae in the aquarium. Nature.

[33] Capoccioni F (2014). The potential reproductive contribution of Mediterranean migrating eels to the *Anguilla anguilla* stock. Deep Sea Res. Part I Oceanogr. Res. Pap..

[34] Boëtius I, Boëtius J (1980). Experimental maturation of female silver eels, *Anguilla anguilla*: estimates of fecundity and energy reserves for migration and spawning. Dana.

[35] Oliveira K, Hable WE (2010). Artificial maturation, fertilization, and early development of the American eel (*Anguilla rostrata*). Can. J. Zool..

[36] Lokman PM, Young G (2000). Induced spawning and early ontogeny of New Zealand freshwater eels (*Anguilla dieffenbachii* and *A. Australis*). New Zeal. J. Mar. Freshw. Res..

[37] Sébert ME (2007). Effects of high hydrostatic pressure on the pituitary-gonad axis in the European eel, *Anguilla anguilla* (L.). Gen. Comp. Endocrinol..

[38] Mikawa Naomi, Yamada Yoshiaki, Horie Noriyuki, Okamura Akihiro, Utoh Tomoko, Tanaka Satoru, Tsukamoto Katsumi (2019). A preliminary experiment regarding the natural induction of gonadal development in female Japanese eels without hormone treatment. Aquaculture Research.

[39] Watanabe S, Minegishi Y, Yoshinaga T, Aoyama J, Tsukamoto K (2004). A quick method for species identification of Japanese eel (*Anguilla japonica*) using real-time PCR: An onboard application for use during sampling surveys. Mar. Biotechnol..

[40] Minegishi Y, Yoshinaga T, Aoyama J, Tsukamoto K (2009). Species identification of *Anguilla japonica* by real-time PCR based on a sequence detection system: a practical application to eggs and larvae. ICES J. Mar. Sci..

[41] Yoshinaga T (2011). Genetic identification and morphology of naturally spawned eggs of the Japanese eel *Anguilla japonica* collected in the western North Pacific. Fish. Sci..

[42] Kagawa H, Tanaka H, Ohta H, Okuzawa K, Iinuma N (1997). Induced ovulation by injection of 17, 20β-dihydroxy-4-pregnen-3-one in the artificially matured Japanese eel, with special reference to ovulation time. Fish. Sci..

[43] Okamura A (2000). Re-examination of the spermatozoal ultrastructure of eels: Observations of the external morphology of spermatozoa in three species. J. Fish Biol..

[44] Okamura A (2007). Effects of water temperature on early development of Japanese eel *Anguilla japonica*. Fish. Sci..

[45] Yamamoto K, Yamauchi K, Kasuga S (1975). On the development of the Japanese eel, *Anguilla japonica* (in Japanese with English abstract). Bulletin of the Japanese Society of Scientific Fisheries.

