# Supplementary material for: Estimation of the spawning time of Japanese eels in the open ocean
Source: Sci Rep. 2020 Mar 6;10:4172. doi: 10.1038/s41598-020-61029-8 (PMC7060335; doi:10.1038/s41598-020-61029-8)
Supplement: Supplementary file 1 — Supplementary information. [file 41598_2020_61029_MOESM1_ESM.pdf]

Supplementary Information

Estimation of spawning time of Japanese eels in open ocean

Takatoshi Higuchi [1,*], Yoshiaki Yamada[2], Shun Watanabe[3], Takahito Kojima[4], Katsumi Tsukamoto[5]

[1]Graduate School of Bioresource Sciences, Nihon University, 1866 Kameino, Fujisawa City, Kanagawa 252-0880, Japan; [2]IRAGO Institute, 377 Ehima-shinden, Tahara, Aichi 441-3605, Japan; [3]Department of Fisheries, Faculty of Agriculture, Kindai University, 3327-204 Nakamachi, Nara 631-8505, Japan; [4]Department of Marine Science and Resources, Nihon University, 1866 Kameino, Fujisawa City, Kanagawa 252-0880, Japan; [5]Graduate School of Agricultural and Life Sciences, The University of Tokyo, 1-1-1 Yayoi, Bunkyo-ku, Tokyo, 113-8657, Japan.

*Corresponding author: Takatoshi Higuchi
Address: Graduate School of Bioresource Sciences, Nihon University, 1866 Kameino, Fujisawa City, Kanagawa 252-0880, Japan
Email: nagoya_g10@yahoo.co.jp

Contents

Suppl. Fig. S1. Times of the collection of Japanese eel 53 of all eggs.

Suppl. Fig. S2. Swimming depth of silver Japanese eels along the West Mariana Ridge.

Suppl. Fig. S3. Photographs of fertilized Japanese eel eggs at different developmental stages.

Suppl. Fig. S4. Dates of the collection of Japanese eel eggs.

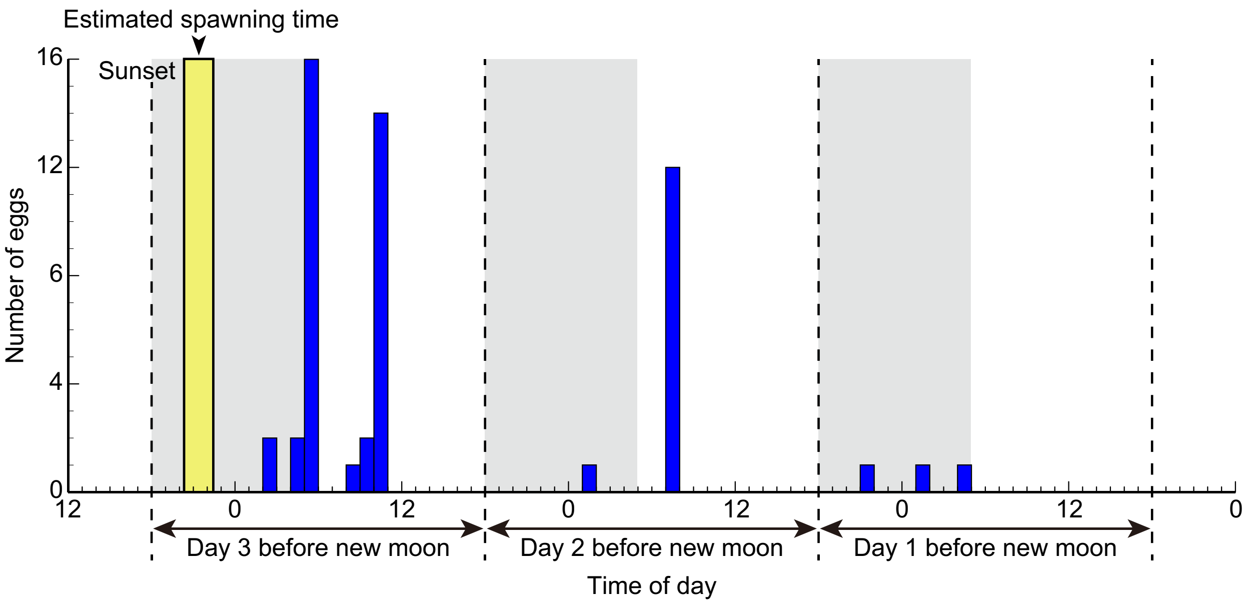

Supplementary Fig. S1. Collection timing of 53 of all 593 collected eggs (shown in Supplementary Fig. S4) that were used for estimation of spawning time. The grey-shaded areas show the time between sunset (dotted vertical line) and sunrise. The peak of spawning time (20:20–22:30 on day 3 before the new moon) is shown by a yellow bar.

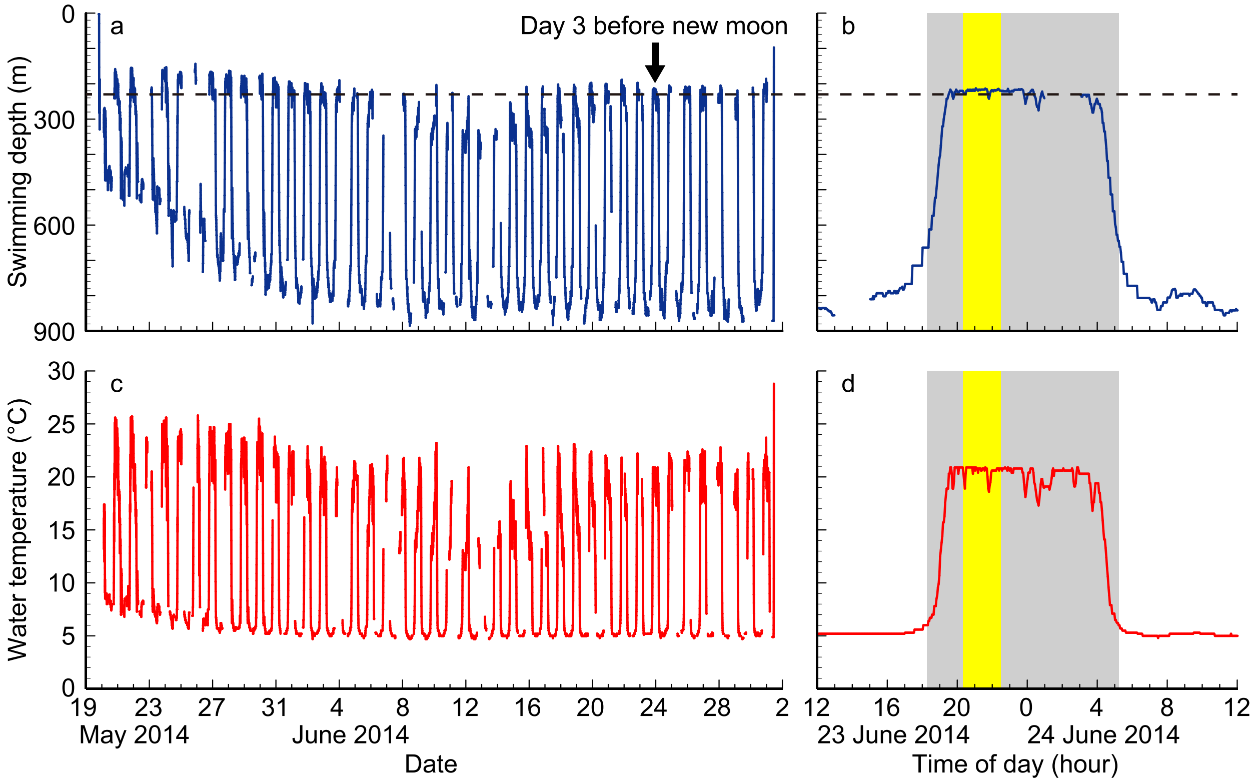

Supplementary Fig. S2. Swimming depth of a silver Japanese eel tagged with pop-up satellite archival tag along the West Mariana Ridge. (**a**) Japanese eels show repeated steady diel vertical migration. The black arrow indicates the swimming depth on day 3 before the new moon[17]. (**b**) Enlarged vertical movement on day 3 before the new moon. The horizontal dotted line indicates the average swimming depth during the period spent swimming in shallow waters at night (229.2 ± 9.9 m). (**c**) The repeated steady daily variation of water temperature experienced during Japanese eel's diel vertical migration[17]. (**d**) Enlarged variation of experienced water temperature on day 3 before the new moon. The shaded area shows the time between sunset and sunrise. The yellow bar shows the time window of estimated spawning time.

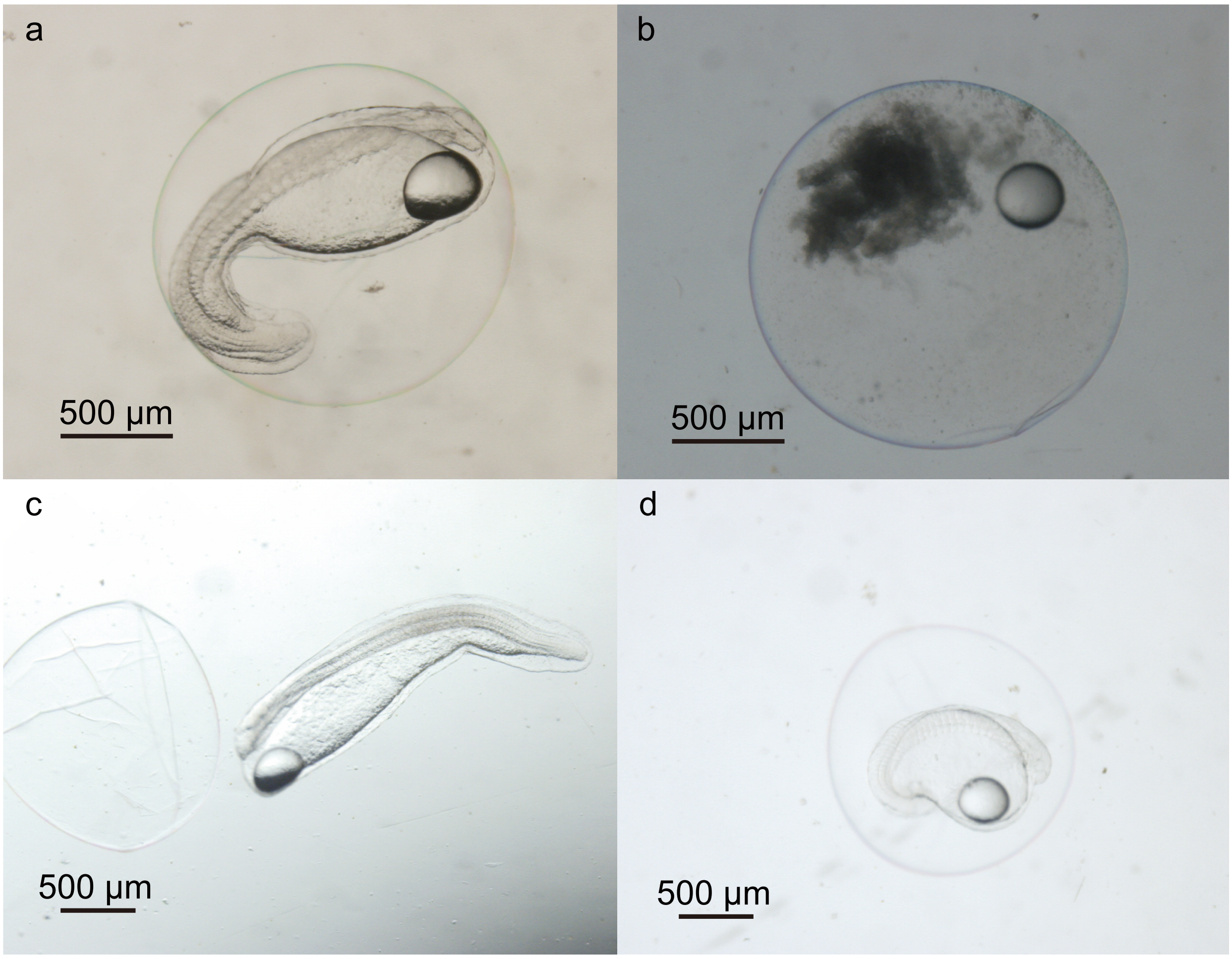

Supplementary Fig. S3. Photographs of fertilised Japanese eel eggs at different developmental stages. (**a**) An egg at the heart formation stage collected at 12°54′N, 141°55′E on 29th June 2011. (**b**) A dead egg collected at 13°5′N, 142°5′E on 29th June 2011. (**c**) A preleptocephalus immediately after hatching. In this case, the egg was collected prior to hatching at 15°5′N, 142°35′E on 19th May 2012, and subsequently hatched during morphological observations. (**d**) An eye and ear vesicle formation stage egg collected at 15°5′N, 142°24′E on 20th May 2012.

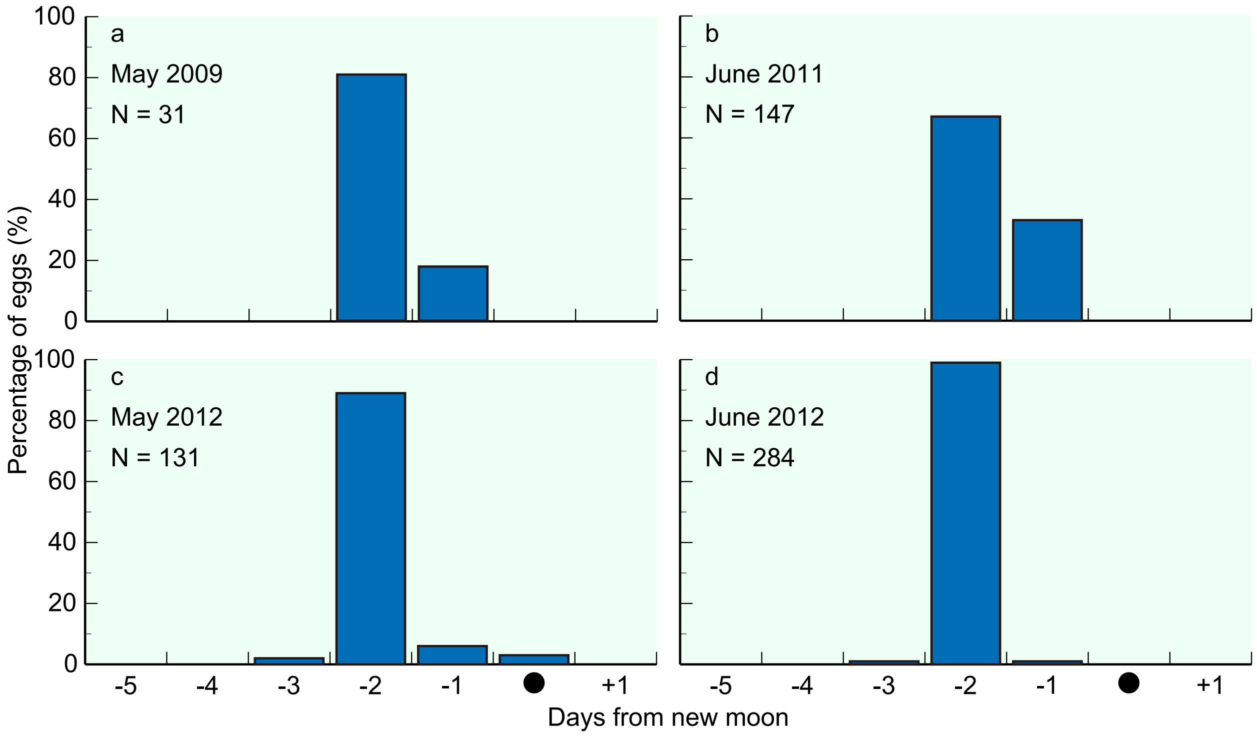

Supplementary Fig. S4. Dates of the collection of Japanese eel eggs in relation to the new moon during each cruise period. A total of 593 eggs of Japanese eels were collected over the course of four cruises in (**a**) May 2009, (**b**) June 2011, (**c**) May 2012, and (**d**) June 2012. All collection dates are plotted in relation to the date of the new moon in each month.
